# Supplementary material for: Improving the Utilization of STRmix™ Variance Parameters as Semi-Quantitative Profile Modeling Metrics
Source: Genes (Basel). 2022 Dec 29;14(1):102. doi: 10.3390/genes14010102 (PMC9859078; doi:10.3390/genes14010102)
Supplement: Supplementary file 1 [file genes-14-00102-s001.zip › Figure S1 and Table S3 (Regression Analysis).pdf]

Supplementary Figure S1

4th-order polynomial regressions of the entire unchallenged data set (2096 samples combining all contributor numbers), including 2-sided 99% confidence intervals, for allele variances (a), reverse stutter variances (b), and forward stutter variances (c). Standard Errors for the Y estimate were calculated in Excel 2016 using the LINEST formula.

(a)

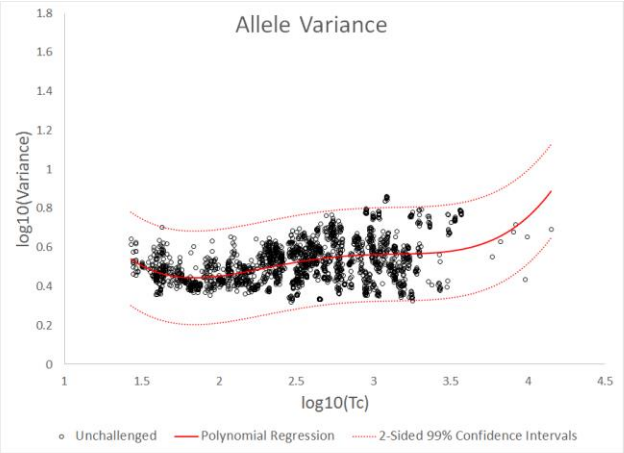

(b)

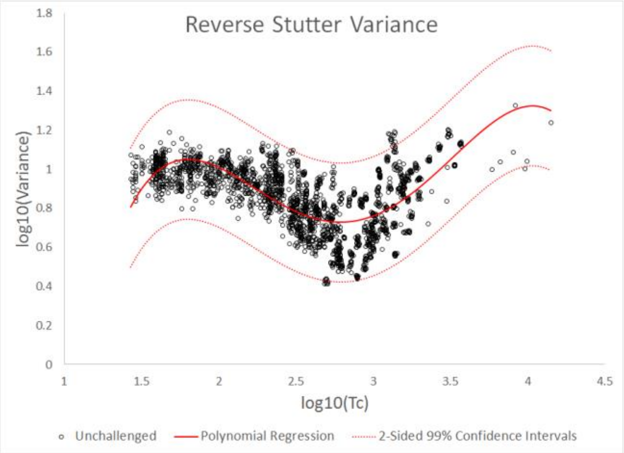

(c)

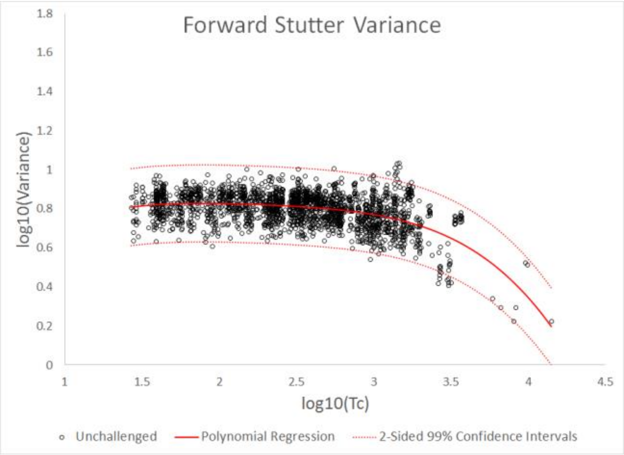

Supplementary Table S3

The overall regression and the individual coefficients were evaluated to see if the variances were significantly better explained by the 4th-order polynomial regression than by a simple straight line with a slope of 0. The general regression line formula is $y = \sum_{i=0}^{4} \beta_i x^i$, where $\beta_{i>0}$ are analogous to the slopes of a straight line formula, and β_0 is analogous to the $y - intercept$. The independent variables (x) were the $\log_{10}(Tc)$ values. The dependent variables (y) were the $\log_{10}(variance)$ values. All calculations were performed in Excel 2016. The β_i regression coefficients and F statistic were calculated using the LINEST formula. F statistics for the overall regressions were significant ($P < 0.01$) for all three variances. T statistics for all allele variance and reverse stutter variance coefficients were significant ($P < 0.01$), and the 99% 2-sided confidence intervals (CIs) for those coefficients never included 0 (analogous to a slope of 0 for $\beta_{i>0}$). For the forward stutter regression, only β_4 was significant ($P < 0.01$), and it was the only coefficient with a 99% confidence interval that did not include 0. These results are consistent with the sample data in Supplemental Figure S1c, where the variance data visually appears uncorrelated with Tc until $\log_{10}(Tc) > 3$.

	Allele	Reverse Stutter	Forward Stutter
Polynomial regression formula	$y = 0.1122x^4 - 1.2206x^3 + 4.8535x^2 - 8.2618x + 5.5312$	$y = -0.2892x^4 + 3.3258x^3 - 13.619x^2 + 23.456x - 13.404$	$y = -0.0348x^4 + 0.313x^3 - 1.0891x^2 + 1.7016x - 0.1678$
R^2	0.1879	0.4774	0.2426
F statistic P value	5.9926E-93	1.1307E-292	1.6337E-124
β_4<br>99% CI<br>T statistic P value	0.1122<br>0.0760 to 0.1485<br>2.3433E-15	-0.2892<br>-0.3355 to -0.2429<br>4.5747E-55	-0.0348<br>-0.0647 to -0.0049<br>0.0027634
β_3<br>99% CI<br>T statistic P value	-1.2206<br>-1.6043 to -0.8369<br>4.0674E-16	3.3258<br>2.8357 to 3.8160<br>4.7026E-64	0.3130<br>-0.0035 to 0.6294<br>0.010849
β_2<br>99% CI<br>T statistic P value	4.8535<br>3.3645 to 6.3426<br>7.8857E-17	-13.6193<br>-15.5216 to -11.7169<br>1.3621E-70	-1.0891<br>-2.3172 to 0.1391<br>0.022342
β_1<br>99% CI<br>T statistic P value	-8.2618<br>-10.7675 to -5.7561<br>3.5300E-17	23.4557<br>20.2544 to 26.6570<br>1.3129E-73	1.7016<br>-0.3651 to 3.7684<br>0.0338971
β_0<br>99% CI<br>T statistic P value	5.5312<br>3.9926 to 7.0698<br>4.5521E-20	-13.4045<br>-15.3702 to -11.4388<br>1.2273E-64	-0.1678<br>-1.4368 to 1.1013<br>0.7332772
