# Supplementary material for: Improving the Utilization of STRmix™ Variance Parameters as Semi-Quantitative Profile Modeling Metrics
Source: Genes (Basel). 2022 Dec 29;14(1):102. doi: 10.3390/genes14010102 (PMC9859078; doi:10.3390/genes14010102)

Supplementary Figure S2

Electropherogram data for the 870 pg 9:1 mixture of cell line DNA that resulted in a 0 LR for the known minor contributor. No overtly aberrant peak data is observed in this mixture, although our regression data indicated that the allele variance parameter for the interpretation of this data was elevated above expectation, given the Tc value.

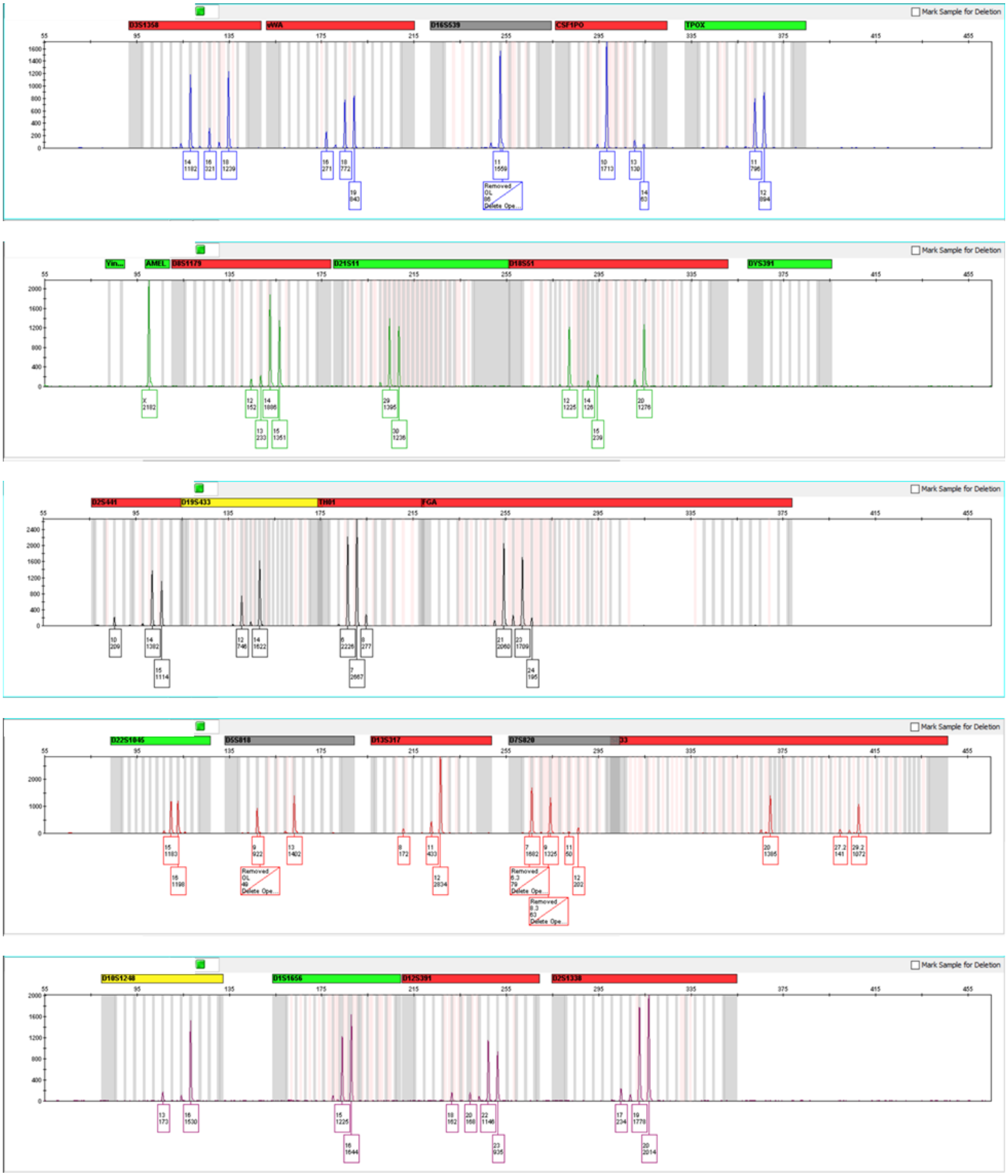

Supplement: Supplementary file 1 [file genes-14-00102-s001.zip › Figure S2 (EPG).pdf]
